# Inhibition of extracellular matrix assembly induces the expression of osteogenic markers in skeletal muscle cells by a BMP-2 independent mechanism

**DOI:** 10.1186/1471-2121-10-73

**Published:** 2009-10-05

**Authors:** Nelson Osses, Juan Carlos Casar, Enrique Brandan

**Affiliations:** 1 Centro de Regulación Celular y Patología, Centro de Regeneración y Envejecimiento (CARE), Departamento de Biología Celular y Molecular, MIFAB, Pontificia Universidad Católica de Chile, Santiago, Chile; 2 Instituto de Química, Facultad de Ciencias, Pontificia Universidad Católica de Valparaíso, Valparaíso, Chile; 3 Departamento de Neurologia, Facultad de Medicina, Pontificia Universidad Católica de Chile, Santiago, Chile

## Abstract

**Background:**

The conversion of one cell type into another has been suggested to be, at the molecular level, the consequence of change(s) in the expression level of key developmental genes. Myoblasts have the ability to differentiate either to skeletal muscle or osteogenic lineage depending of external stimuli. Extracellular matrix (ECM) has been shown to be essential for skeletal muscle differentiation, through its direct interaction with myoblasts' cell receptors. We attempt to address if ECM also plays a role in the osteogenic differentiation of skeletal muscle cells.

**Results:**

Inhibition of proteoglycan sulfation by sodium chlorate in myoblast cultures strongly affects ECM synthesis and deposition and induces the expression of the osteogenic lineage markers alkaline phosphatase (ALP) and osteocalcin in mononuclear cells. Induction of ALP by sodium chlorate does not affect the expression of specific muscle determination transcription factors, such as MyoD and Myf-5, in the same cells. The osteogenic transcription factor Cbfa-1 expression is also unaffected. Induction of ALP is not inhibited by a soluble form of BMP receptor IA. This suggests that the deviation of the myogenic pathway of C2C12 myoblasts into the osteogenic lineage by inhibitors of proteoglycan sulfation is BMP-2 independent. The increase of osteogenic markers expression can be totally prevented by an exogenous ECM. Interestingly, a similar BMP-2-independent ALP activity induction can be observed in myoblasts cultured on an ECM previously synthesized by BMP-2 treated myoblasts. Under *in vivo *conditions of increased ECM turn-over and deposition, as in the *mdx *dystrophic muscle and during skeletal muscle regeneration, an induction and relocalization of ALP is observed in a subpopulation of skeletal muscle fibers, whereas in normal skeletal muscle, ALP expression is restricted to blood vessels and some endomysial mononuclear cells.

**Conclusion:**

These results suggest that signals arising from the ECM induce the expression of osteogenic markers in muscle cells by a mechanism independent of BMP-2 and without affecting the expression of key muscle or osteogenic determination genes. An induction and relocalization of ALP is also observed in *mdx *and regenerating skeletal muscles, *in vivo *conditions of increased muscle ECM deposition or turnover.

## Background

Understanding the cellular and molecular basis of cell-determination and terminal differentiation is important as to gain insight into the mechanisms of normal development and, potentially, for the achievement of successful stem cell-based therapies. The observation that embryological commitments can be reversed or erased under certain circumstances, in a phenomenon known as metaplasia [1], is particularly interesting.

Skeletal muscle cells are a helpful model for studying cell commitment and differentiation. During skeletal muscle development, fusion of mononuclear myoblasts to form multinucleated myotubes is a central event. This process is partially controlled by the sequential expression of some regulatory proteins, the myogenic regulatory transcription factors (MRFs) of the MyoD family (MyoD, Myf-5, myogenin and MRF4). Forced expression of MRFs in different mesenchymatic cell lines can induce their transdifferentiation into skeletal muscle [2,3]. The expression and activity of these master genes are regulated by several polypeptide growth factors as well as by retinoic acid [4-7]. The presence of extracellular matrix (ECM) is critical for a proper skeletal muscle differentiation. For instance, inhibitors of collagen synthesis have been shown to inhibit myoblast differentiation [8,9]. Addition of either RGDS peptides or antibodies against integrin receptor to myoblast cultures has also a strong inhibitory effect on muscle differentiation [10,11]. We have shown that inhibitors of proteoglycan synthesis, such as sodium chlorate and β-D-xylosides, produce a strong inhibition of ECM assembly that is followed by repression of skeletal muscle differentiation [11,12], even though the MRF myogenin is expressed and properly localized at the nuclei. This inhibition can be totally rescued by the addition of an exogenous ECM, suggesting that the ECM and its receptors provide an appropriate and permissive environment for lineage-specific cell differentiation [11].

Studies on stem cells transplantation have highlighted the role of local tissue signals for specific cell-type determination, but the relative contribution of intrinsic or genetic signals and extrinsic or ECM signals in cell behavior are not completely understood. Within skeletal muscle tissue specific cells exhibit apparent stem-cell like plasticity [13-16]. BMP-2 treatment of the mouse myoblast cell line C2C12 [17] and muscle satellite cells isolated from adult mice [18] inhibits myotube formation and induces the expression of alkaline phosphatase activity (ALP) and osteocalcin, changing their differentiation pathway into the osteoblastic lineage. Interestingly, in several muscular diseases [19-21] and animal models for skeletal muscle dystrophy [22], the level of ALP is increased. We have studied microenvironmental changes of skeletal muscle in the *mdx *mouse, an animal model of Duchenne muscular dystrophy (DMD) [23], finding an important increase in the amount of ECM proteoglycans present at endomysium and perimysium [24-26]. We have also found an up-regulation of proteoglycans during the process of damage-induced muscle regeneration [27,28].

In this paper, we show that inhibition of proteoglycan sulfation by sodium chlorate in myoblast cultures induces the expression of osteogenic lineage markers and that this can be prevented by the presence of an exogenous ECM. ECM synthesized by BMP-2 treated-myoblasts can also induce ALP in myoblasts. This induction is mediated by BMP-2 independent mechanisms in both cases. Expression of osteogenic markers does not affect the expression of muscle commitment MRFs or the osteogenic determination gene Cbfa-1. We finally show that in *mdx *and regenerating skeletal muscles, *in vivo *conditions of increased muscle ECM deposition or turnover, an induction and relocalization of ALP was found.

## Results

### Inhibition of Proteoglycan Synthesis in Myoblasts Induces the Expression of Osteogenic Lineage Markers

**Figure 1:**
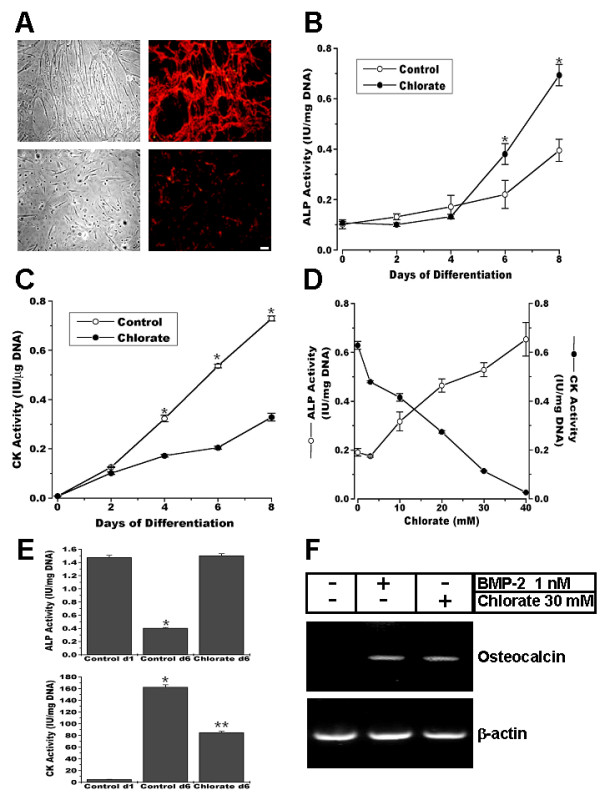
**Inhibition of proteoglycan sulfation induces osteogenic markers expression in C2C12 myoblasts**. Myoblasts were induced to differentiate in the absence (control) or presence of 30 mM sodium chlorate (chlorate) **A**. Phase contrast microscopy (left column) and perlecan indirect immunofluorescent staining (right column) of non-permeabilized cells C2C12 cells at 6 days of differentiation under control (upper row) and chlorate (lower row) conditions. Bar = 25 μm. **B and C**. ALP (**B**) and CK (**C**) activities were measured at different time points after inducing differentiation of C2C12 in control or chlorate conditions. Both enzymatic activities were significantly different in control vs. chlorate (p < 0.0001, unpaired t-test). **D**. ALP and CK measurements at eight days after induction of differentiation of C2C12 cells under different concentrations of sodium chlorate. **E**. ALP (upper panel) and CK (lower panel) activity determinations at day 1 and 6 of differentiation of primary cultures of muscle cells from rat fetal hind limbs under control or chlorate conditions. * = p < 0.001 different from Control d1 (lower panel) or Control d1 and Chlorate d6 (upper panel); ** = p < 0.001, different from Control d1 and d6 (One-way Analysis of Variance (ANOVA) followed by Tukey-Kramer multiple comparisons test). All the data are presented as Mean ± S.D. of two (E) or three (B-D) independent experiments performed in triplicate. **F**. RT-PCR for Osteocalcin and β-actin performed on RNA extracted from C2C12 cells at 6 days of differentiation in the absence or presence of 1 nM BMP-2 or 30 mM sodium chlorate.

Sodium chlorate is a specific inhibitor of proteoglycan sulfation which does not affect cell protein or DNA content [12,29]. Table 1 shows that in cultures of the clonal myoblastic cell line C2C12, sulfation of proteoglycans is strongly inhibited by sodium chlorate 30 mM. We have shown that sodium chlorate treatment affects the deposition of different ECM components such as laminin, fibronectin and ECM proteoglycans [11,12]. Cultures of control C2C12 myoblasts induced to differentiate and stained with anti-perlecan antibody show a bright and fibrillar specific staining of this ECM heparan sulfate proteoglycan (Figure 1A, *top*). Conversely, sodium chlorate treatment of parallel cultures almost abolished the perlecan staining (Figure 1A, *bottom*). Figure 1A also shows that when myoblasts were induced to differentiate in the presence of sodium chlorate, a marked inhibitory effect on both the number and length of the myotubes was observed, with a concomitant increment in the number of remaining mononuclear cells (around 50% of increase, data not shown). Due to the known capacity of C2C12 cells to generate cells from different lineages under special culture conditions, the expression of ALP, an early marker of osteogenic differentiation, was evaluated in cultures treated with sodium chlorate. ALP activity was determined during myogenic differentiation in the presence or absence of ECM as a consequence of sodium chlorate treatment. Figure 1B shows that around a 100% of increase in ALP activity is observed in myoblasts under sodium chlorate treatment as compared to control cultures at day 8 of differentiation. Under similar conditions, the induction of creatine kinase (CK) activity, a skeletal muscle differentiation marker, was diminished to less than 50% of control values by the absence of ECM (Figure 1C). Figure 1D shows that both the induction of ALP and the inhibition of CK were totally dependent of the concentration of sodium chlorate present in the differentiation medium. To evaluate if the inductive effect on ALP activity by sodium chlorate treatment was a particular phenomenon of this clonal cell line, primary cultures of rat muscle myoblasts were prepared and incubated under differentiation conditions [30]. Figure 1E (top panel) shows that ALP activity was inhibited 6 days after the culture cells were induced to differentiate. In contrast, cultures in the presence of sodium chlorate maintained high values of ALP activity. High values of ALP, probably arising from intramuscular connective tissue cells, have been previously observed in primary cultures of skeletal muscle cells under growth conditions [31]. Bottom panel of Figure 1E shows that primary cultures of muscle cells differentiate appropriately *in vitro *as evaluated as the induction of CK activity, which was inhibited by sodium chlorate treatment as was seen in C2C12 myoblasts.

**Table 1:** Effect of Chlorate on [^35^S]-SO_4 _Incorporation into Proteoglycans and Glycosaminoglycans in C2C12 Myoblasts.

	**cpm/mg DNA**	
	
	**Control**	**Chlorate**
		
Medium		
Proteoglycans	3950 ± 520	240 ± 40
Glycosaminoglycans	1990 ± 570	520 ± 110
		
Extracts		
Proteoglycans	11180 ± 550	820 ± 20
Glycosaminoglycans	3820 ± 460	290 ± 100

C2C12 cells were incubated for 48 hours with 30 mM sodium chlorate. Sulfate incorporation during the last 18 hours was measured in medium and detergent extracts. [^35^S]Sulfate radioactivity for total (proteoglycans plus glycosaminoglycans) and trichloroacetic acid-precipitated material (proteoglycans) samples, spotted on filters containing the cationic detergent cetyl pyridinium chloride **(**CPC), was measured with liquid scintillation counting. DNA content was measured in same aliquots of detergent extracts (13.3 μg/μl for control and 18.0 μg/μl for chlorate treated cells). The specific activity values correspond to the means ± SD obtained from three different plates.

To further characterize the phenotype of myoblast differentiation in the presence of sodium chlorate, the expression of the specific osteogenic differentiation marker osteocalcin was studied by RT-PCR. Figure 1F shows that sodium chlorate treatment of C2C12 cells under differentiation conditions induces the expression of osteocalcin mRNA, indicating their osteogenic nature. The same Figure shows, as a positive control, the induction of osteocalcin by recombinant BMP-2 as has been previously described [17,32].

**Figure 2:**
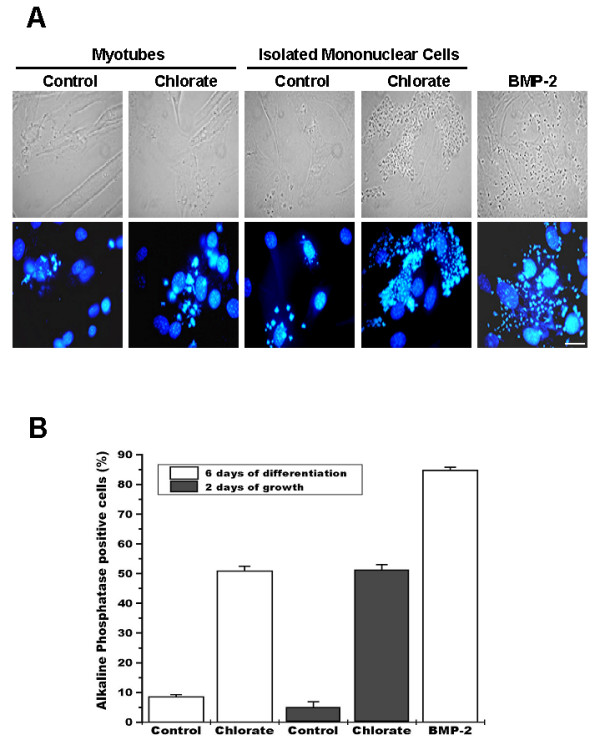
**Under conditions of proteoglycan sulfation inhibition, alkaline phosphatase activity is present in mononuclear skeletal muscle cells**. **A**. C2C12 cells were induced to differentiate for 6 days in the absence (first column) or presence of 30 mM sodium chlorate (second column). Remaining mononuclear cells from control (third column) or sodium chlorate treated cultures (fourth column) were isolated and grown for 48 hours. C2C12 cells induced to differentiate in the presence of 5 nM BMP-2 were used as positive control (fifth column). Unpermeabilized cells were fixed and alkaline phosphatase activity was visualized as a fluorescent precipitate using ELF-97 detection kit; nuclear staining was performed with 1 μg/ml Hoechst 33258 (lower row). Phase contrast microscopy is shown in the upper row. Bar = 25 μm. **B**. Percentage of ALP-positive mononuclear cells in the different experimental conditions described in A. Values correspond to mean ± S.E.M. of 10 different microscopy fields from two independent experiments. ANOVA analysis followed by Tukey-Kramer multiple comparisons test shows there are no statistically significant differences when both control or both chlorate conditions (p > 0.05) are compared. All other comparisons are significantly different (p < 0.001).

To visualize in which cell type the observed induction of ALP activity was localized, C2C12 cultures were induced to differentiate in the presence or absence of the sulfation inhibitor and stained for ALP activity. As shown in Figure 2A only mononuclear cells are expressing ALP activity. A quantitative analysis indicates that under differentiation conditions in the presence of sodium chlorate, almost 50% of the total mononuclear cells expressed ALP activity (Figure 2B). When these mononuclear cells were isolated, replated and maintained under growth conditions, ALP activity remained higher in cultures that were treated with sodium chlorate as compared to cells isolated from control cultures. Induction of ALP activity by BMP-2 treatment is shown as a positive control in the same Figure.

These results suggest that the inhibition of proteoglycan sulfation by sodium chlorate affects ECM assembly and induces the expression of osteogenic markers in mononuclear muscle cells concomitant with an inhibition of the process of skeletal muscle differentiation.

### Induction of ALP Activity by Inhibitors of Proteoglycan Sulfation Does Not Affect the Expression of Specific Muscle Transcription Factors of Commitment

**Figure 3:**
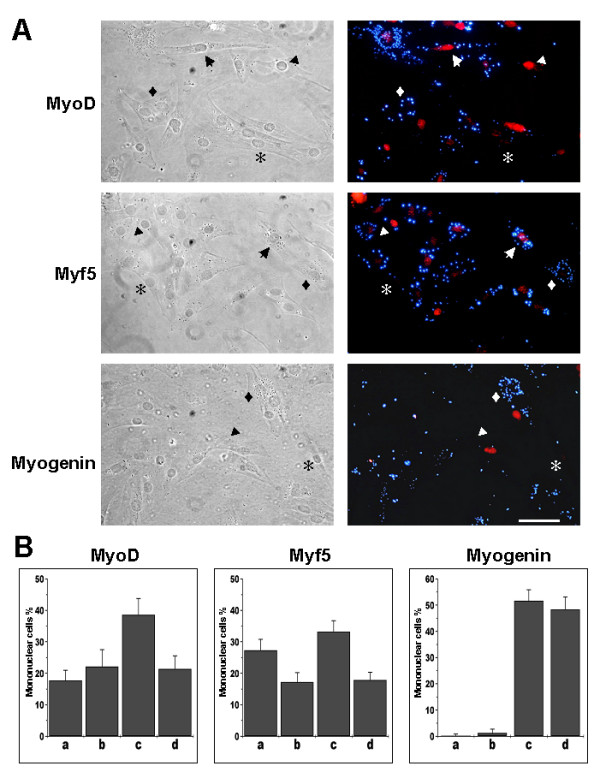
**Alkaline phosphatase positive mononuclear cells express myogenic regulatory factors**. Mononuclear cells were isolated from C2C12 cell cultures after 6 days of differentiation in presence of 30 mM sodium chlorate. Permeabilized cells were fixed and stained for alkaline phosphatase activity using ELF-97 detection kit and for MRFs using polyclonal anti-mouse MyoD, anti-human Myf5 or anti-rat myogenin antibodies. TRITC-conjugated anti-rabbit IgG was used as secondary antibody. Symbols represent: arrow, MRF+/ALP+; arrowhead, MRF+/ALP-; diamond, MRF-/ALP+; asterisk, MRF-/ALP-. Bar = 50 μm. **B**. Quantification of single or double labeled cells was performed in 10 independent fields. a, MRF+/ALP+; b, MRF+/ALP-; c, MRF-/ALP+; MRF-/ALP-. (Mean ± S.D. of two independent experiments).

It is well known that skeletal muscle differentiation is under the control of MRFs [33]. Proliferative C2C12 myoblasts express the muscle determination factors MyoD and Myf5. Under serum deprival conditions myogenin expression is triggered and terminal skeletal muscle differentiation is induced. To evaluate if the cells expressing ALP activity did not express MRFs, mononuclear cells were isolated from cultures after 6 days of differentiation in the presence of sodium chlorate. Figure 3A shows ALP activity and localization of MyoD, Myf-5 and myogenin in monuclear cells. It can be seen that some mononuclear cells, as recognized in phase contrast visualization co-express ALP and MyoD or Myf5 (top and middle row). Quantitative analysis indicates that about 30 to 40 % of total mononuclear cells express ALP without expression of Myo or Myf5 (Figure 3B). The same figure shows that around 20% of the cells express MyoD or Myf5 without expressing ALP activity but also 20 to 30% of the mononuclear cells co-express ALP activity and MyoD or Myf5. Isolated mononuclear cells express very little levels of myogenin, as expected. These results indicate that the induction of ALP activity under conditions of a deficient ECM in mononuclear cells is not dependent on a turning off of the expression of determination MRFs.

**Figure 4:**
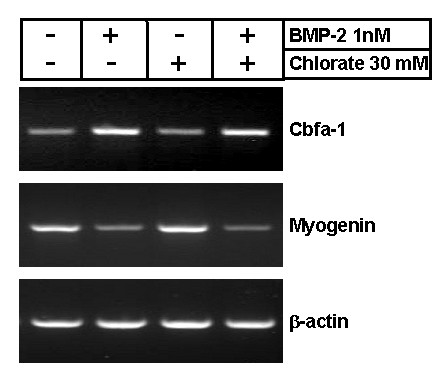
**Cbfa-1 expression is not induced by inhibition of proteoglycan sulfation**. RNA from C2C12 cells after 6 days of differentiation in the absence or presence of 1 nM BMP-2 or 30 mM sodium chlorate was obtained. After reverse transcription using Oligo-dTs, Cbfa-1, myogenin and β-actin cDNAs were amplified using specific primers. The reactions were performed within the linear range for both time and RNA quantity.

We also evaluated the expression of Cbfa-1, an specific osteogenic determination gene which induces osteoblastic differentiation, by RT-PCR [34,35]. Presence of Cbfa-1 has been observed in myogenic muscle cells derived from satellite cells as well as C2C12 myoblasts [36,37]. Figure 4 shows low levels of Cbfa-1 expression in myoblasts triggered to differentiate. Cbfa-1 expression is induced by BMP-2 but not by sodium chlorate treatment. As a comparison the same Figure shows that myogenin is present in myoblasts induced to differentiate and that it is inhibited by BMP-2, but is unaffected by sodium chlorate treatment. As mentioned before, the inhibitory effect of sodium chlorate on skeletal muscle differentiation was independent of myogenin expression and its nuclear localization [11,12]. These results indicate that sodium chlorate treatment of skeletal muscle cells does not affect the expression of osteogenic or muscle key differentiation genes.

### Induction of ALP Activity by Sodium Chlorate Treatment is BMP-2 Independent

**Figure 5:**
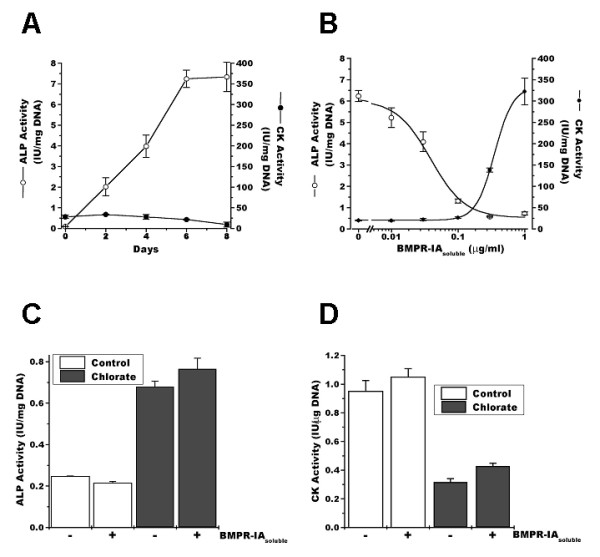
**Alkaline phosphatase activity induced by inhibition of proteoglycan sulfation is not BMP-2 dependent**. **A**. C2C12 cells were induced to differentiate in the presence of 5 nM BMP-2. ALP (open circles) and CK (closed circles) activity was determined as a function of time. The mean ± S.D. of two independent experiments performed in triplicate is presented. **B**. C2C12 cells were induced to differentiate in the presence of 5 nM BMP-2 and different concentrations of soluble BMP receptor IA (BMPR-IA_soluble_). ALP (open circles) and CK (closed circles) activity was determined at day 6. Values presented as mean ± S.D. of two independent experiments performed in triplicate. C2C12 cells were induced to differentiate either in the absence (control) or presence of 30 mM sodium chlorate (chlorate) or 1 μg/ml soluble BMP receptor IA (BMPR-IA_soluble_). ALP (**C**) and CK (**D**) activity was determined at day 8 days. The mean ± S.E.M, of two independent experiments performed in triplicate is presented. ANOVA analysis followed by Tukey-Kramer multiple comparisons test shows that there are no statistically significant differences (p > 0.05) within the control or chlorate conditions. All other comparisons are significantly different (p < 0.001).

To evaluate if the observed induction of ALP in C2C12 by sodium chlorate was dependent of BMP-2 expression, we studied the effect on this phenomenon of adding a soluble form of BMP receptor IA (BMPR-IA). Figure 5A shows that when C2C12 myoblasts were treated with BMP-2, ALP activity was induced whereas CK activity was strongly inhibited. Figure 5B shows that the addition of the BMPR-IA blocked almost completely the induction of ALP activity produced by BMP-2 with a concomitant induction of CK activity on the sixth day of differentiation. Figure 5C shows that 1 μg/ml BMPR-IA, the highest dose studied, has no effect either on the induction of ALP activity or on the inhibition of CK by sodium chlorate treatment (Figure 5D). These results suggest that the deviation of the myogenic differentiation pathway of C2C12 myoblasts into the osteogenic lineage by sodium chlorate is BMP-2 independent.

### Exogenous ECM Prevents Osteogenic Markers Expression Induced by Inhibition of Proteoglycan Sulfation

**Figure 6:**
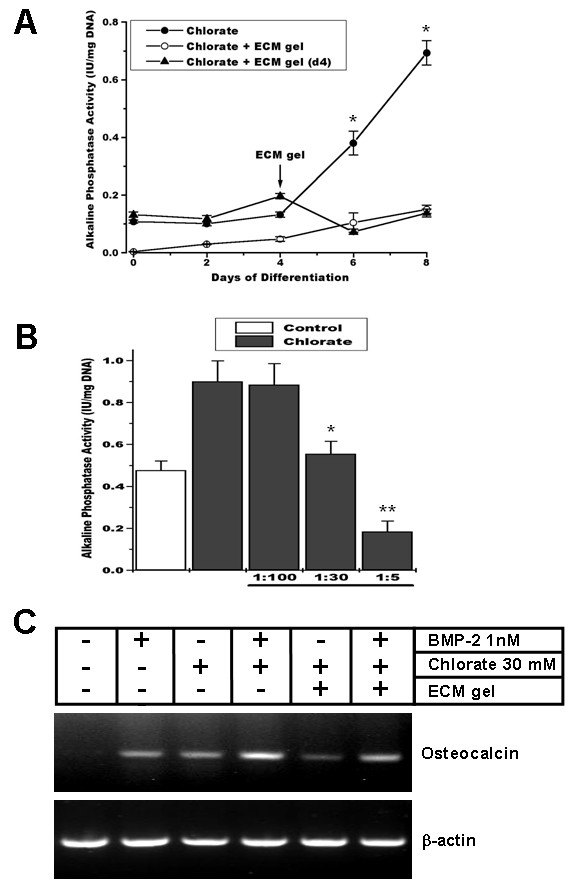
**Addition of exogenous ECM prevents osteoblastic marker expression induced by inhibition of proteoglycan sulfation**. **A**. C2C12 cells were induced to differentiate in the presence of 30 mM sodium chlorate (closed circles). ECM gel was added at day 0 (open circles) or at day 4 of differentiation (closed triangles). ALP activity was determined at different time points. Data are presented as mean ± S.E.M. of two independent experiments performed in triplicate (p < 0.0001, unpaired t-test between chlorate and ECM gel addition). **B**. ALP activity was determined in C2C12 cells induced to differentiate for 8 days in the absence (control) or presence of 30 mM sodium chlorate (chlorate) and different ECM gel dilutions were added to the cell culture. Data are presented as mean ± S.E.M. of two independent experiments performed in triplicate. * = significantly different from chlorate without and with 1:100 ECM gel, p < 0.05; ** = significantly different from all the other conditions p < 0.05 (ANOVA followed by Tukey-Kramer multiple comparisons test between chlorate conditions). **C**. RNA was extracted from C2C12 cells after 6 days of differentiation in the presence or absence of 30 mM sodium chlorate, 1 nM BMP-2, and/or ECM gel. After reverse transcription using Oligo-dTs the cDNA was amplified using specific primers for osteocalcin and β-actin.

We have previously shown that the inhibitory effect of sodium chlorate on skeletal muscle differentiation was the result of an altered deposition of ECM by the muscle cells as a consequence of inhibition of proteoglycans sulfation [11,12]. We now evaluated if it was possible to prevent the induction of ALP activity produced by sodium chlorate treatment by providing the cells with exogenous ECM. In the experiments shown in Figure 6, the effect of ECM gel, a basement membrane-like ECM obtained from mouse EHS sarcoma, on the induction of ALP activity was studied. Figure 6A shows that the ECM gel prevented the induction of ALP activity. This effect was observed by adding the ECM-gel at the beginning of the differentiation (day 0) or at day 4 of differentiation. Figure 6B shows a titration of the inhibitory effect of ECM-gel on the induction of ALP by sodium chlorate. Interestingly, the ECM gel at higher concentration was able to inhibit ALP activity below the levels observed for control cells, as shown in Figure 6B. The inhibitory effect of ECM-gel on the induction of osteogenic markers as consequence of sodium chlorate treatment was also observed on the induction of osteocalcin, as shown in the Figure 6C. This Figure also shows that co-treatment of myoblasts with BMP-2 and sodium chlorate augments the effect on osteocalcin induction, although its enhanced expression could not be totally prevented by the addition of the ECM-gel. These results suggest that the induction of osteogenic markers by inhibition of proteoglycan sulfation can be prevented by an exogenous ECM.

### ECM from BMP-2 Treated Myoblasts Induces ALP Activity in muscle cells

**Figure 7:**
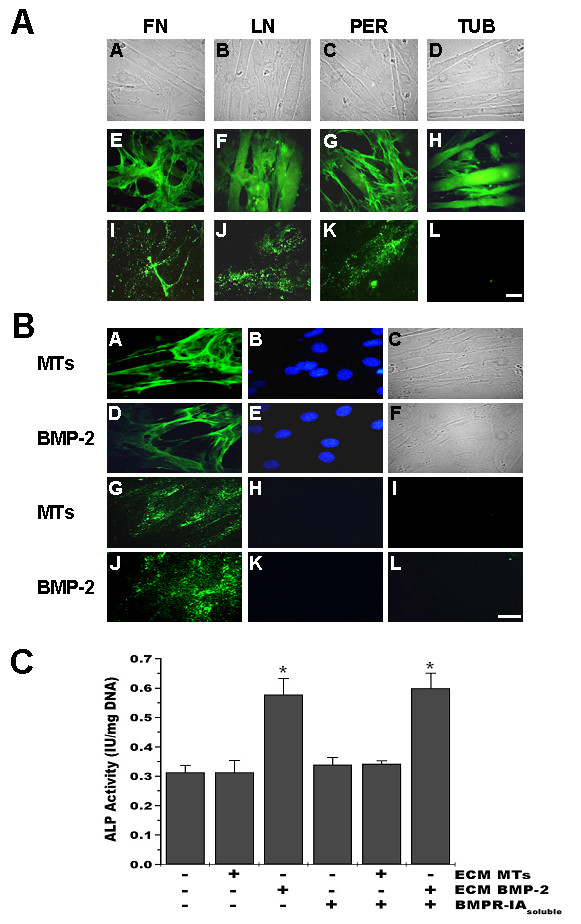
**ECM produced by BMP-2 treated myoblasts induces alkaline phosphatase in C2C12 cells**. **A**. C2C12 cells were induced to differentiate for 6 days. Unpermeabilized cells (E-G) or coverslips after cell removal by EDTA treatment (I-K) were stained with anti-fibronectin (FN), anti-laminin (LN) or anti-perlecan (PER) antibodies. H and L show anti-tubulin (TUB) staining after permeabilization. FITC-conjugated secondary antibodies were used. Phase contrast microscopy is shown (A-D). Bar = 25 μm. **B**. C2C12 cells were induced to differentiate for 6 days in the absence (MTs) or presence of 5 nM BMP-2 (BMP-2). Unpermeabilized cells (A, D) or ECM (G, J) obtained as described in A were stained with anti-fibronectin antibodies. FITC-conjugated secondary antibodies were used. Nuclear staining was performed with 1 μg/ml Hoechst 33258 (B, E, H, K). Phase contrast microscopy for cells is shown (C and F) and staining without the primary antibody is shown in I and L. Bar = 25 μm. **C**. C2C12 cells were plated on ECM from myotubes (ECM MTs) or BMP-2 treated cells (ECM BMP-2), as described above, and induced to differentiate in the absence or presence of 1 μg/ml soluble BMP receptor IA (BMPR-IA_soluble_). ALP activity was determined at day 6. Data are presented as mean ± S.E.M. of three independent experiments performed in triplicate. * = significantly different from the other values, but not between them, p < 0.015 (ANOVA followed by Tukey-Kramer multiple comparisons test).

The above described experiments suggest that the inductive effect of sodium chlorate on osteogenic markers expression is likely a consequence of an altered ECM. We explored then if this ECM modification was specific for the induction of osteogenic markers expression in muscle cells or if it would be possible to produce a similar effect using ECM obtained from BMP-2 treated myoblasts. To evaluate this point we plated muscle cells over ECM obtained from cultured myoblasts induced to transdifferentiate by BMP-2 treatment. Figure 7A shows that after removal of the cells by PBS/EDTA incubation, the ECM of differentiated myoblasts remained attached to the culture plates as can be seen by the presence of some ECM components. The same is true if myoblasts are treated with BMP-2 (Figure 7B). The effect of these ECMs on ALP activity was evaluated by plating C2C12 myoblasts on them and inducing skeletal muscle differentiation. Figure 7C shows that myoblasts plated on ECM obtained after BMP-2 treatment presented a significant increase in ALP activity. This increase was not prevented by the addition of BMPR-IA during the differentiation process. This Figure also shows that when myoblasts were induced to differentiate on ECM isolated from myotubes, there was no induction of ALP activity. These results suggest that the ECM synthesized as consequence of BMP-2 treatment of myoblasts has the ability to induce ALP activity by a BMP-2 independent mechanism.

### Induction and Cellular Relocalization of ALP Activity in a Dystrophic Animal Model

**Figure 8:**
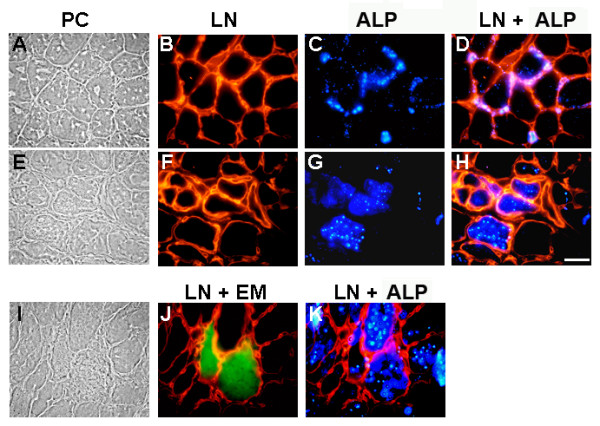
**Expression of alkaline phosphatase by skeletal muscle fibers of the *mdx *mouse**. Cross-sections of diaphragm from control (A-D) and *mdx *(E-K) 16 weeks old mice were stained with anti-laminin for basal lamina labeling and detected with rhodamine-conjugated secondary antibodies (B, F), and with ELF-97 detection kit for ALP activity (C, G). Merge of ALP (blue) and basal lamina staining (red) from the same fields is shown (D, H). In panel J, the merge from cross-sections of diaphragm from *mdx *16 weeks old mice stained with anti-embryonic myosin detected with fluorescein-conjugated secondary antibodies (green) and with anti-laminin detected with rhodamine-conjugated secondary antibodies (red) is shown. Merge of ALP staining (blue) and basal lamina staining (red) from the same fields is shown (K). Phase contrast is shown at the left (A, E, I). Bar = 25 μm.

**Figure 9:**
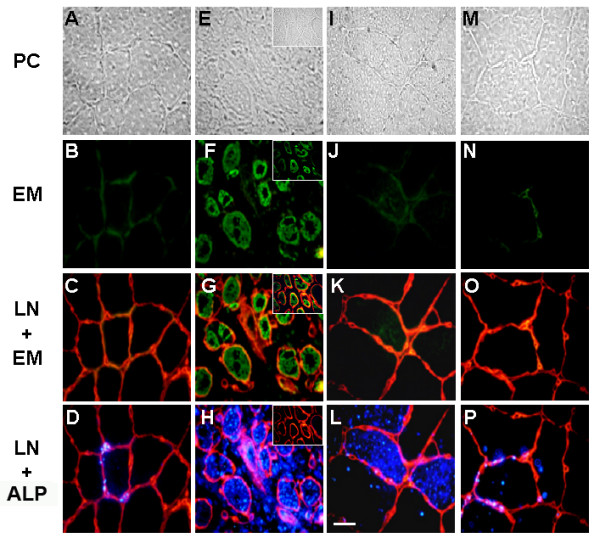
**Expression of alkaline phosphatase by skeletal muscle fibers during skeletal muscle regeneration**. Cross-sections from control TA (A-D), and after five (E-H), fifteen (I-L) and twenty eight (M-P) days after barium chloride injection were stained with anti-embryonic myosin antibodies (B, F, J, N) and detected with fluorescein-conjugated secondary antibodies. Merge of embryonic myosin staining (green) and laminin staining (red), as described in Figure 7, is shown (C, G, K, O). Merge of ALP staining using ELF-97 detection kit (blue) and laminin staining (red) from the same fields is shown (D, H, L, P). Phase contrast is shown at the top (A, E, I, M). Different fields showing ALP-negative/embryonic myosin-positive fibers five days after the injection are presented as inserts (E-H). Bar = 25 μm.

It has been shown that human dystrophic muscle presents an augmented ALP activity [19]. ALP activity was evaluated in control and *mdx *mouse diaphragm sections, where significant changes in amount and composition of the ECM occur [24-26]. Figure 8 shows that in normal muscle (A-D) ALP activity is localized at the endomysium, as is evidenced by laminin staining, and that it is specifically associated to cells and vascular structures surrounding individual myofibers (Figure 8B). In *mdx *muscles, in contrast, ALP activity was found mainly within muscle fibers as shown in Figure 8G-H. In both control and *mdx *muscle, ALP activity was only found in restricted areas spread throughout the tissue's cross section, either in the endomysium surrounding a limited number of fibers, or in a limited number of fiber groups, respectively. It is well known that *mdx *muscles are under continuous degeneration-regeneration cycles. To evaluate if the re-localization of ALP in the diseased muscle was a consequence of the formation of new fibers, we evaluated, by double staining, the expression of both ALP and embryonic myosin (EM), a marker of regenerating fibers. Figure 8(I-K) shows that no strict spatial relationship between ALP activity and EM positive fibers was found, as ALP(+)/EM(+), ALP(+)/EM(-) and ALP(-)/EM(+) fibers were observed. Hence, the increase and relocalization of ALP activity appears not to be strictly linked to the formation of new fibers. However, as different time courses in the expression of these two markers, or the invasion of degenerating myofibers by ALP(+) mononuclear cells are potential confounding factors for the interpretation of these results, we evaluated this point further by studying the localization of ALP activity in a model of damage-induced muscle regeneration in control mice. This model shows more synchronized skeletal muscle fibers formation than the *mdx*. Intramuscular injection of a 1.2% barium chloride solution induces necrosis of most skeletal muscle fibers of the muscle in the first 3-4 days, which is followed by the complete regeneration of the tissue [38]., Extensive remodeling of the ECM and changes in the expression of its constituents occur during the process [39,28]. Figure 9 shows that, as was observed in the diaphragm, ALP activity is localized in some of the cells in the endomysium of normal Tibialis Anterior muscle. Five and 15 days after damage induction ALP activity is observed inside some of the newly formed fibers. However, other regenerating fibers from the same section at day five, positive for EM (Figure 9F*insert*), were negative for ALP staining (Figure 9H*insert*). After 15 days of damage induction, EM expression was silent whereas ALP was still present inside some fibers (Figure 9J-L) and after 28 days, ALP was again mainly localized in the endomysium around individual fibers (Figure 9P). These results suggest that under conditions of tissue regeneration and ECM remodeling, ALP expression is found in a subpopulation of regenerating skeletal muscle fibers, in contrast to normal conditions, where ALP activity in skeletal muscle is restricted to blood vessels and some interstitial mononuclear cells in the endomysium.

## Discussion

Metaplasia, the conversion of one cell type into another, has been suggested to be, at the molecular level, the consequence of a change in the expression of key developmental genes. In normal development, particular combinations of these master genes are activated in each embryo region following the expression of local inducing signals. Postnatally, numerous examples of pathologic metaplasia are known, but the underlying mechanisms are poorly understood [40,41]. Metaplasias are nearly always associated with situations of tissue regeneration, in the absence of many of the signals that are present during development. In this study we have demonstrated that when myoblasts are cultured under conditions that affect or modify ECM synthesis and assembly, an important and significative increment in the expression of osteogenic markers is observed. This occurs through a mechanism that did not involve either the turn-off or turn-on of master genes such as Myf-5, MyoD, myogenin or Cbfa-1. The expression of these osteogenic markers was fully reverted by the addition of an exogenous ECM. Furthermore, we show that the ECM produced by myoblasts induced to transdifferentiate into osteoblasts by BMP-2 treatment was able to induce ALP activity in C2C12 myoblasts under normal skeletal muscle differentiation conditions. Both phenomena seem to be BMP-2 independent because the induction of osteogenic markers was not inhibited by competence with a soluble form of the BMP-2 receptor ectodomain. These results suggest that signals arising from the ECM induce the expression of osteogenic markers in myoblasts and emphasizes that a proper ECM is required for correct skeletal muscle differentiation.

We have previously shown that skeletal muscle differentiation was strongly inhibited under conditions where the assembly of the ECM was affected by inhibitors of proteoglycans synthesis as sodium chlorate or β-D-xyloside [11,12]. As a consequence of these treatments, a decrease in focal adhesion kinase (FAK) phosphorylation was observed [11]. This enzyme is activated upon interaction of integrins with ECM constituents [42,43], but interference of this interactions by the addition of RGDS peptides affects normal skeletal muscle differentiation [10,11]. These observations indicate that a proper interaction and signaling between cells and their environment is crucial for adequate terminal differentiation.

Myoblasts are considered fully committed to muscle differentiation due to the expression of the MRFs Myf-5 and MyoD. We have previously shown that, under differentiation conditions, proper expression and the nuclear localization of the MRF myogenin is not sufficient to drive a successful myogenesis and that myoblasts-ECM interactions are also required [11]. Here we show that when myoblasts are grown in the absence of an appropriate ECM, they express ALP and osteocalcin concomitant to the expression of skeletal muscle determination-associated MRFs in the same cells. Therefore down-regulation of MyoD or Myf-5 is not necessary for the appearance of an osteoblastic-like phenotype. Furthermore we show that the induction of Cbfa-1, a transcription factor essential for osteogenesis [34,35], is not required for the initial expression of osteogenic markers in skeletal muscle cells. Our observation is coincident with a previous report showing that ALP induction after BMP-2 treatment of mouse myogenic cells preceded the down-regulation of MyoD expression, and that Cbfa-1 was normally expressed in committed myogenic cells [36]. All these evidences support the proposed stock options model of differentiation in skeletal muscle cells, where multiple determination genes can be expressed and depending on the differentiation-inducing signals the cells follow a terminal differentiation pathway [36].

Several examples of transdifferentation, the conversion of one differentiated cell type to another, are known in the literature. Myoblasts can be made to transdifferentiate into adipocytes after introduction of the transcription factors C/EBPα and peroxisome-proliferator-activated receptor (PPAR)-γ [44]. It is also known that C2C12 myoblasts can be converted to adipocytes after transfection with a dominant-negative version of the transcription factor TCF4 [45] and, as mentioned before, myoblasts can differentiate into osteoblasts by BMP-2 treatment [17]. Although these observations suggest that muscle cells preserve multi-potentiality, the exact requirements to achieve the different phenotypes are still not clear. It is well known that the MRF MyoD will convert several cell lines into muscle [2], but, as discussed above, the forced expression of a particular gene might not be sufficient to drive metaplasia without the proper environmental signals.

It has been shown that BMP-2/4 associated to the ECM are essential for differentiation of osteoblastic cells [46]. The fact that a soluble BMP-2 receptor ectodomain was unable to inhibit the induction of osteogenic markers in myoblasts cultured in a deficient ECM indicates that we are in presence of a different mechanism of osteogenic induction. Likely, changes in or the absence of some type of signals between the ECM and the cells, presumably through integrins, are sufficient to trigger an osteoblastic phenotype. This concept is reinforced by the fact that the ECM obtained from BMP-2 treated myoblasts was able to induce ALP activity in normal myoblasts. If this is the result of a new type of interaction between ECM and its cell surface receptors or of the absence of some other interactions requires further investigation. We have experimental data that indicate that the proteoglycan population synthesized by BMP-2 treated myoblasts is different in composition [47], but changes in other ECM constituents may also have a role. It has been shown that fibronectin supports the induction of ALP by ascorbic acid in fibroblasts through its interaction with integrin α5β1, whereas type I collagen fibrils cause the suppression of ALP expression [48]. However, the possibility that BMP-2 may be inducing the expression of another osteogenic factor, such as BMP-7, which could be retained at the ECM can not be excluded from our experiments.

Heterotopic bone formation within skeletal muscle is a widely observed pathologic phenomenon, specially after repeated trauma, but it is exacerbated in rare genetic diseases such as Fibrodysplasia Ossificans Progressiva [45]. Osteoprogenitor cells are thought to reside in skeletal muscle, although their identity in the tissue has not been clearly determined [49]. On the other hand, it is known that diseased muscles show increased ALP activity, as in DMD, facioscapulohumeral dystrophy, polymyositis, etc. [20,21,50]. As *mdx *skeletal muscle is under constant rounds of degeneration-regeneration and shows enhanced ECM remodeling and deposition [24], we thought it would be an appropriate model to evaluate muscle ALP expression in an *in vivo *situation. We found not only an increase in ALP activity but a relocalization of the activity in dystrophic muscle, in contrast to normal mice where the ALP was localized in scattered cells and blood vessels around individual fibers. In *mdx *muscle ALP activity was localized within muscle fibers that formed small groups in the tissue. Expression of ALP activity by skeletal muscle fibers had been previously reported in DMD and congenital muscular dystrophy (CMD) muscle biopsies, and its expression was suggested to be localized in immature fibers [19]. We evaluated if there was a correlation between the relocalization of ALP activity and the formation of new muscle fibers. We found that although some new muscle fibers, determined by the expression of EM, were positive for ALP this was not always the case and EM-positive fibers containing no ALP activity were also found. When ALP expression during skeletal muscle regeneration was evaluated, we also found ALP activity within a small number of newly formed myotubes. ALP expression in muscle fibers lasted for longer than the expression of embryonic myosin, but one month after the induction of damage ALP activity was found again localized in the endomysium. It is known that during skeletal muscle regeneration, fusing myoblasts are in contact with a scaffold of remnant ECM from degenerated fibers before they begin to synthesize their own ECM [38,39]. Considering that muscle tissue possess cells with osteogenic potential that may also act as muscle precursor cells [31,36] and our findings of ALP expression by endomysial cells in normal muscle and ALP induction in groups of fibers during muscle regeneration, it is interesting to speculate whether this phenomenon, if exacerbated by repetition in time or by impairment of its regulatory mechanisms, may be related to heterotopic ossification.

The induction and relocalization of ALP activity found under these two *in vivo *experimental conditions of skeletal muscle differentiation and ECM remodeling, extend our observations of co-expression of differentiation markers for distinct lineages and suggest that they are perhaps a consequence of a different interaction between the muscle fibers and the surrounding ECM. This concept is supported by observations of ALP increase in the dystrophic muscle of dy/dy mouse [22] and the presence of positive myofibers for ALP in CMD [19], phenotypes that lack of laminin α2 expression, so the normal interaction between the basement membrane and the dystrophin-glycoprotein complex or integrins is missing [23].

It is known from cell transplantation studies that cells of different origins, such as bone marrow [51] and neural tissue [52], can be incorporated into skeletal muscle. Furthermore, muscle-derived cells different from satellite cells, like the side population of dissociated muscle cells or cells present in the interstitial space can differentiate into muscle and hematopoietic cells or muscle and endothelial cells, respectively [53-55]. In this work we have shown that C2C12 myoblasts can be induced to express osteogenic markers by signals from the ECM. The mechanisms of metaplasia of these different cell populations are not clear, but what it might be critical *in vivo *is the fact that these cells can become reprogrammed or prepared to acquire different fates when surrounded by a particular environment.

## Conclusion

The conversion of one cell type into another has been suggested to be, at the molecular level, the consequence of change(s) in the expression level of key developmental genes. ECM has been shown to be essential during skeletal muscle differentiation, through direct interaction with myoblasts cell receptors. Myoblasts have the ability to differentiate either to skeletal muscle or osteogenic lineage depending of external stimuli. We explored the possibility that ECM plays a role in the change of differentiation pathway of skeletal muscle cells into osteogenic cells.

We show that the inhibition of proteoglycan sulfation by sodium chlorate in myoblast cultures induces the expression of osteogenic lineage markers that can be prevented by the addition of an exogenous ECM. ECM synthesized by BMP-2 treated-myoblasts can also induce ALP in myoblasts. This induction is mediated by BMP-2 independent mechanisms in both cases. Expression of osteogenic markers does not affect the expression of muscle commitment MRFs or the osteogenic determination gen Cbfa-1. We finally show that in *mdx *and regenerating skeletal muscles, *in vivo *conditions of increased muscle ECM turn-over and deposition, an induction and relocalization of ALP was found from its expression by mononuclear cells to a sub-group of regenerating muscle fibers.

These results suggest that signals arising from the ECM induce the expression of osteogenic markers in muscle cells by a mechanism independent of BMP-2 without affecting the expression of key muscle or osteogenic determination genes. The induction and relocalization of ALP was also observed in *mdx *and regenerating skeletal muscles, *in vivo *conditions of increased muscle ECM deposition or turnover.

## Methods

### Cell Cultures

The mouse cell line C_2_C_12 _derived from regenerating adult leg skeletal muscle (American Type Culture Collection) was grown and induced to differentiate as described [56,57]. Sodium chlorate (final concentration 30 mM) was added to the cultures at the time of plating. ECM gel (Sigma Chem. Co., St. Louis, MO) was added after 2 days of growth when the cells were switched to differentiation medium. The medium was removed and 30 μl/cm^2 ^of ECM gel (diluted 1:5 in DME-Ham's F12) was added over the cells and allowed to polymerize for 2 hours at 37°C. Fresh differentiation medium was then added to the plates containing the polymerized gel. BMP-2 was a generous gift from Genetic Institute (Cambridge, MA). Soluble BMPR-IA corresponding to the extracellular domain of mouse BMPR-IA was purchased from R& D Systems Inc. (Minneapolis, MN), and was changed every two days at the moment of medium renewal.

Primary cultures of myoblasts were obtained from the hind limb muscles of 18-day-old rat embryos and cultured as previously described [30]. 3 × 10^5 ^cells were plated on 35 mm plastic tissue culture dishes coated with 1% gelatin and maintained in MEM-199 medium containing 10% (v/v) horse serum, 10 units/ml penicillin and 100 g/ml streptomycin.

### Animals and Experimental Muscle Injury

Parental strains of control (C57BL/10) and *mdx *(C57BL10 *mdx*/*mdx*) mice were obtained from Jackson Laboratories (Bar Harbor, ME, USA). The animals were kept at room temperature with a 24 hour night-day cycle and fed with pellets and water *ad libitum*. Injury of normal Tibialis Anterior muscle (TA) was performed by barium-chloride injection as described previously [28,38]. All protocols were conducted under strict accordance and with the formal approval of the Animal Ethics Committee of the P. Universidad Católica de Chile.

### Fluorescence Microscopy

Cells to be immunostained were grown on glass coverslips. The medium was removed and the plates were rinsed with PBS. For staining of extracellular proteins the cells were incubated with primary antibodies for 1 hour at 4°C before fixation (perlecan 1:1500, fibronectin 1:100 and laminin 1:100). After rinsing, the cells were fixed with 3% paraformaldehyde for 30 minutes at room temperature. For staining of intracellular proteins the cells were fixed with paraformaldehyde and then permeabilized with 0.05% Triton X-100 in PBS. The cells were rinsed with TSB (2% BSA in tris-buffered saline) and then incubated for 1 hour at room temperature with the primary antibodies (myogenin 1:50, MyoD 1:25, Myf-5 1:25). For detection, permeabilized and not permeabilized cells were incubated for 30 minutes at room temperature with affinity purified fluorescein- or rhodamine-conjugated secondary antibodies (PIERCE, Rockford, IL) diluted in TSB. After immunofluorescence alkaline phosphatase activity was detected using ELF-97 detection kit (Molecular Probes, Inc., Eugene, OR) according to the manufacturer directions. For nuclear staining, fixed cells were incubated 5 minutes in 1 μg/ml Hoechst 33258 in PBS. After rinsing, the cover slips were mounted with fluorescent mounting medium (Dako Corporation, CA). Fluorescence was visualized using a Nikon Eclipse microscope equipped for epifluorescence. Fields from the same experiment were photographed and treated under identical conditions. Polyclonal anti-mouse MyoD, anti-human Myf-5 and anti-rat myogenin were from Santa Cruz Biotechnology (Santa Cruz, CA). Polyclonal anti-human fibronectin was purchased from Sigma and anti-mouse perlecan was a generous gift from Dr. John R. Hassell.

For immunohistochemistry, cryostat sections (6 μm) of control or *mdx *muscle, or TA harvested at different times after BaCl_2 _injection were fixed for 20 minutes in 3% paraformaldehyde in phosphate-buffered saline (PBS), pH 7.4, blocked with 3% BSA in PBS and incubated at 4°C overnight with primary antibodies anti-embryonic myosin (1:100) or/and anti-laminin (1:100). Sections were then washed and incubated respectively with either anti-mouse-FITC or anti-rabbit-TRITC conjugated secondary antibodies (all diluted 1:100, Pierce, IL) for 1 hour at room temperature. Alkaline phosphatase activity was detected as described before. Polyclonal anti-mouse laminin was purchased from Sigma and monoclonal anti-human embryonic myosin F1.652 was developed by Dr H. Blau [58] and obtained from the Developmental Studies Hybridoma Bank, developed under the auspices of the NICHD and maintained by The University of Iowa, Department of Biological Sciences, Iowa City, IA.

### Analysis of enzymatic activities

Myoblasts and myoblasts induced to differentiate were washed twice with PBS, lysed by incubation with PBS containing 0.1% Triton X-100 for 10 minutes at 4°C and harvested by scraping. ALP activity was quantified using ρ-nitrophenyl phosphate in a pH 10.1 buffer as a substrate. Formation of ρ-nitrophenol as a function of time at 37°C was determined at 405 nm. CK activity was determined using the CPK assay kit (Sigma Chem. Co., St. Louis, MO).

### RT-PCR analyses

Total RNA was isolated from cell cultures using Trizol (Invitrogen Corporation, Carlsbad, Ca). Equal amounts of RNA were reverse-transcribed with Ready To-Go You-Prime First-Strand Beads kit (Amersham Biosciences Inc., Piscataway, NJ) using oligo-dT as primers. For PCR the paired primers for mouse osteocalcin used were CTC TGA CCT CAC AGA TGC CAA/ACT TGC AGG GCA GAG AGA GAG G (332 base pair); for mouse Cbfa-1 were TTT GCC CTC ATC CTT CAC TCC/GAA AGC AAA TCT TGG GCA ATA(541 base pair); for mouse myogenin were TCA CAT AAG GCT AAC ACC CAG/GCA AAA CCA CAC AAT GCT TAG T (503 base pair); and for mouse β-actin were ATG GAT GAC GAT ATC GCT G/ATG AGG TAG TCT GTC AGG T (568 base pair). Samples were denatured at 94°C for 5 minutes, followed by amplification rounds consisting of denaturing at 94°C for 30 seconds, annealing at 60°C for 30 seconds and extension at 72°C for 30 seconds for 35 cycles and 72°C for 10 minutes.

### Extracellular Matrix Preparation

C2C12 cells were induced to differentiate in 2.5 % horse serum in the presence or absence of 5 nM BMP-2 on 6-well plates (Costar, Corning, NY). At day 6 the cell layer was detached with 5 mM EDTA in PBS at 37°C for 10 minutes [59,60]. Following cell removal the underlying ECM was gently rinsed five times with cold PBS and used immediately for cell plating.

### DNA determination

DNA was determined in aliquots of cell extracts according to the method of Labarca and Paigen [61]. Briefly, the fluorescence of Hoechst 33258 bound to DNA was measured and aliquots compared to known concentrations of calf thymus DNA, which was used as a standard.

## Authors' contributions

NO carried out al the experiments using sodium chlorate. JCC did the experiments involving the normal and dystrophic mice. EB conceived the study, and participated in its design and coordination and drafted the manuscript. All authors read and approved the final manuscript.
